# Integrating linkage mapping and comparative transcriptome analysis for discovering candidate genes associated with salt tolerance in rice

**DOI:** 10.3389/fpls.2023.1065334

**Published:** 2023-01-24

**Authors:** Leiyue Geng, Wei Zhang, Tuo Zou, Qi Du, Xiaoding Ma, Di Cui, Bing Han, Qixing Zhang, Longzhi Han

**Affiliations:** ^1^ Institute of Crop Sciences, Chinese Academy of Agricultural Sciences, Beijing, China; ^2^ Institute of Coastal Agriculture, Hebei Academy of Agriculture and Forestry Sciences, Tangshan, China; ^3^ Tangshan Key Laboratory of Rice Breeding, Tangshan, China

**Keywords:** rice, salt stress, linkage mapping, RNA-Seq, candidate gene

## Abstract

Salinity is one of the most widespread abiotic stresses affecting rice productivity worldwide. Understanding the genetic basis of salt tolerance is key for breeding salt-tolerant rice varieties. Numerous QTLs have been identified to help dissect rice salt-tolerance genetic mechanisms, yet only rare genes located in significant QTLs have been thoroughly studied or fine-mapped. Here, a combination of linkage mapping and transcriptome profiling analysis was used to identify salt tolerance-related functional candidate genes underlying stable QTLs. A recombinant inbred line (RIL) population derived from a cross between Jileng 1 (salt-sensitive) and Milyang 23 (salt-tolerant) was constructed. Subsequently, a high-density genetic map was constructed by using 2921 recombination bin markers developed from whole genome resequencing. A total of twelve QTLs controlling the standard evaluation score under salt stress were identified by linkage analysis and distributed on chromosomes 2, 3, 4, 6, 8 and 11. Notably, five QTL intervals were detected as environmentally stable QTLs in this study, and their functions were verified by comparative transcriptome analysis. By comparing the transcriptome profiles of the two parents and two bulks, we found 551 salt stress-specific differentially expressed genes. Among them, fifteen DEGs located in stable QTL intervals were considered promising candidate genes for salt tolerance. According to gene annotations, the gene *OsRCI2-8*(*Os06g0184800*) was the most promising, as it is known to be associated with salt stress, and its differential expression between the tolerant and sensitive RIL bulks highlights its important role in salt stress response pathways. Our findings provide five stable salt tolerance-related QTLs and one promising candidate gene, which will facilitate breeding for improved salt tolerance in rice varieties and promote the exploration of salt stress tolerance mechanisms in rice.

## 1 Introduction

Rice (*Oryza sativa* L.) is a premier staple food for more than half of the world’s population, and soil salinity is a major abiotic stress hindering rice production because it reduces rice yield and limits agricultural land utilization (Munns and Tester, 2008). Rice is usually considered a salt-sensitive crop, and the normal growth of conventional cultivars is seriously affected when the soil salinity exceeds 6 dS/m (Kumar et al., 2013). Therefore, more salt tolerant cultivars are required. Genetic improvement is an economical and effective way to improve salt tolerance in rice (Qin et al., 2020). Salt tolerance in rice is a quantitative trait controlled by multiple genes with a complex genetic mechanism, and it is difficult to improve salt tolerance using traditional breeding methods (Liu et al., 2022). Modern molecular breeding strategies such as marker assisted-selection (MAS), transgene and gene editing can accelerate the process of selecting for salt-tolerant varieties and improve breeding efficiency (Chen et al., 2021). However, it is necessary to identify quantitative trait loci (QTLs) or clone the key genes controlling salt tolerance (Ganie et al., 2021).

Linkage mapping is a classical strategy that is powerful enough to dissect complex quantitative traits (Xu et al., 2017). Over the past two decades, remarkable efforts have been made to identify salt tolerance-related QTLs in rice (Jing and Zhang, 2017). Based on information from the Gramene database (https://archive.gramene.org/qtl/ ) and supplementary literature (Jing and Zhang, 2017), more than one thousand salt tolerance-related QTLs have been discovered and are evenly distributed on all 12 rice chromosomes. The dissection of the genetic basis of tolerance to salinity has provided useful information for understanding the possible mechanism of salt tolerance (Ganie et al., 2019).

Although salt-tolerant QTLs are numerous, only rare QTLs showing significant effects have been thoroughly studied or map-based cloned. For example, *qSKC-1* and *qSNC-7* are involved in regulating K^+^/Na^+^ homeostasis under salt stress and explain 48.5% and 40.1%, respectively, of the total phenotypic variance (Lin et al., 2004). Isolation of the *qSKC-1* gene by map-based cloning revealed that it encodes a member of the HKT-type transporter family (Ren et al., 2005). Subsequently, *DST*, which encodes a zinc-finger transcription factor that negatively regulates salt tolerance and controls stomatal closure by directly modulating genes related to H_2_O_2_ homeostasis, was isolated by mutant and map-based cloning (Huang et al., 2009b). *HST*, which encodes a B-type response regulator and negatively regulates salt tolerance, was cloned by MutMap (Takagi et al., 2015). *qSE3*, which encodes a K^+^ transporter gene, *OsHAK21*, promotes seed germination and seedling establishment under salinity stress in rice (He et al., 2019). Compared to the number of mapped QTLs, the number of cloned genes is very small. There is a large gap between discovering salt-tolerant QTLs and effectively exploring functional salt-tolerant genes.

In our opinion, there are at least three reasons for this gap: (1) some QTLs, which were only identified in a single study with a minor LOD and contribution rate, may not be completely reliable and should be further validated. (2) The majority of reported QTLs are minor QTLs and may not be stably detected across multiple environments. It was difficult to identify functional genes by traditional map-based cloning. (3) Most of the reported QTLs have been identified based on low-density markers, such as SSRs and RFLPs, with large confidence intervals containing too many genes. These markers are not able to provide precise and complete information about the numbers and locations of the QTLs controlling salt tolerance. It is imperative to identify reliable major QTLs by combining ultra-high-density genetic maps and precise phenotypic data across multiple environments.

In the current paper, we utilized an ultra-high-density genetic map that could rapidly anchor major genes to improve the precision of QTL positioning and narrow the range of candidate genes. Moreover, we evaluated the salt tolerance of RILs across two environments and three years to obtain convincing results. As a result, we have attained a few stable and major QTL intervals controlling salt tolerance. However, the linkage mapping strategy does not usually provide an accurate way to distinguish candidate genes underlying the QTLs without further fine-mapping (Bohra et al., 2020). Positional cloning of QTLs may be an effective approach for the identification of genes underlying target QTLs, but this is still a laborious, time-consuming task (Takeda and Matsuoka, 2008). An alternative method is to screen target QTL intervals for physically proximate genes with annotations or gene ontologies reflecting the trait of interest. However, hundreds of candidate genes usually reside in target QTLs, and it is still very difficult to predict which of these genes is actually responsible for the functional polymorphism leading to trait differences.

RNA-Seq, which uses next-generation sequencing technology to profile transcriptomes and detect differentially expressed genes (DEGs), has been employed to characterize the responses of various plant species to environmental stresses (Urano et al., 2010). RNA-Seq analysis has shown high resolution, sensitivity, and reproducibility (Ozsolak and Milos, 2011). Recently, a strategy integrating linkage mapping and RNA-Seq was developed that can mutually verify functional chromosome regions or genes and can accurately identify the potential candidate genes residing in target QTLs. Through a platform combining QTL mapping and RNA-Seq analysis, four candidate genes were colocalized with QTLs for salt tolerance (Wang et al., 2017). In that paper, six candidate genes related to anaerobic germination tolerance in rice were revealed by combining QTL mapping and RNA-Seq (Yang et al., 2019). Kong et al. (2021) integrated GWAS, QTL, mapping and RNA-Seq to identify candidate genes for seed vigor in rice (*Oryza sativa* L.) and successfully predicted seven promising candidates associated with seed vigor. The combined use of linkage mapping with transcriptome profiling represents a practical solution to further refine the mapping resolution and identify potential candidate genes.

The identification of genes for quantitative traits is difficult when using any single approach due to the complex inheritance patterns of the traits and limited resolving power of the individual techniques. The aim of the present study was to identify promising candidate genes for salt tolerance in rice by combining genetic mapping and transcriptome profiling of bulked RILs with extreme phenotypes.

## 2 Materials and methods

### 2.1 Plant materials

An RIL population containing 253 lines was developed by crossing the salt-tolerant japonica cultivated variety Jileng 1 (P1) as the donor and the salt-sensitive indica cultivated variety Milyang 23 (P2) as the receptor using the single seed descent (SSD) method (**Figure S1**). The F_2_ generation from Jileng 1×Milyang 23 was subjected to more than ten rounds of self-pollination to generate the RIL population.

Twenty of the most tolerant (T) and most sensitive (S) RILs of the Jileng 1×Milyang23 mapping population were identified based on the consistency and performance of SES across three years (2017, 2018 and 2019) to create the two bulks. Two bulks along with the parents were planted under salt stress and normal conditions and prepared for RNA-Seq analysis. The parents under salt stress and normal conditions were named SalP1, SalP2, NorP1, and NorP2, respectively. Similarly, the bulks under salt stress and normal conditions were named SalT, SalS, NorT and NorS, respectively.

### 2.2 Phenotyping for SES under saline

#### 2.2.1 Experimental design

Phenotyping for SES was performed on stabilized F10 plants of 253 RILs along with two parents and five control varieties. During the middle of June to late July from 2017 to 2019, each independent trial was carried out under two environments (greenhouse and paddy field, **Figure S2**) on the salt identification base of HAAFS, Tangshan, China (39° 20’N, 118° 17’E), respectively. The seedlings were arranged by an α-lattice design (**Table S2**) and transplanted into two environments (greenhouse and paddy field) at the 3-leaf stage, with 2 to 4 independent replicates.

#### 2.2.2 Field management and salt treatment

In the greenhouse, the transplantation area was 15 ×10 cm, with 10 holes in each plot in a single row and a single seedling in each hole. In the field, the transplantation area was 25×13 cm, with 30 holes in each plot in 6 rows and a single seedling in each hole. All seedlings were planted on the same day in conventionally tilled plots. Field management practices were performed according to the most followed agricultural practices of local farmers. The nitrogen (N), phosphorus (P), and potassium (K) fertilizers, in the form of urea, single superphosphate, and murate of potash, were applied at rates of 120, 60, and 60 kg/ha, respectively.

The salt stress condition consisted of applying underground brine to the established seedings starting at 7 days after transplanting, and the conductivity of the irrigated water layer was adjusted to 10 ds/m. The normal condition consisted of applying fresh water with a conductivity less than 1 ds/m. Five samples were collected every day to measure the conductivity of the irrigated water layer. If the conductivity of the irrigated water layer increased or decreased due to water evaporation or rainfall, fresh water or underground brine was adjusted.

#### 2.2.3 Phenotypic evaluation

Salt injury symptoms, such as leaf drying and burning, stunted plant growth, reduced tillering, and leaf tip drying and withering, were perceptible in the stressed environments. The overall phenotypic performance of the population under salt stress was reflected by visual SES scores. After two and four weeks of salt stress, plants were scored following the standard protocol of IRRI with some modifications (Gregorio et al., 1997). W2SES and W4SES corresponded to standard evaluation score of two and four weeks after salt treatment, respectively. The SES of RILs under field conditions were assigned to five grades, 1, 3, 5, 7, and 9, corresponding to extremely tolerant, tolerant, moderately tolerant, moderately sensitive, and sensitive, respectively (**Figure S3**). The SES of RILs in the greenhouse was scored as 10 contiguous plants per line and calculated by the modified standard evaluation score in the plot as follows (Geng et al., 2020):


$$\begin{array}{l} \text{SES = }\sum \left(\text{number of salt-injured plants at all levels × salt evaluation score}\right)\text{/total}\;\\ \text{number of plants under investigation} \end{array}$$

### 2.3 DNA and RNA extraction, genotyping by sequencing, and SNP identification

#### 2.3.1 DNA extraction, sequencing and SNP calling

DNA was extracted from the two parents and 253 RILs at the F10 generation using the CTAB method and quantified using both a NanoDrop 2000c Spectrophotometer and 1% agarose gel electrophoresis. Sequencing was performed on the Illumina HiSeq2500 platform to generate 150 bp paired-end reads (Novogene Bioinformatics Technology Co., Ltd, China). In this study, Jileng 1 and Milyang 23 were sequenced to ~40× coverage, while RILs were sequenced to ~5× coverage. The primary sequencing datasets generated during the current study are available in the Genome Sequence Archive, https://ngdc.cncb.ac.cn/gsa (accession no. CRA008901).

Filtering of the low-quality data was performed to produce better-quality mapping. The clean data were then aligned to the Nipponbare reference genome (Os-Nipponbare-Reference-IRGSP-1.0, http://plants.ensembl.org/Oryza_sativa) (Kawahara et al., 2013) using BWA software (Li et al., 2009). The calculations of the sequencing coverage and depth were performed using Samtools (Li and Durbin, 2009). Then, the Genome Analysis Toolkit (GATK) (Mckenna et al., 2010) was used to detect the SNPs with default parameters. High-quality SNPs with a minimum sequencing depth of 3 for each RIL and a quality score of 30 were selected for further analysis.

#### 2.3.2 RNA extraction, transcriptome profiling and qRT–PCR

Under both salt stress and normal conditions, after approximately 14 days of treatment, leaf samples of the parents and each line within the tolerant and sensitive bulks were collected and immediately stored in liquid nitrogen for RNA extraction. Total RNA was extracted using a Sigma Spectrum Plant Total RNA Kit (Sigma–Aldrich, St. Louis, MO, USA). RNA samples were reverse-transcribed to cDNA using the High-Capacity cDNA Archive Kit (Applied Biosystems, Foster City, California, USA). Sequencing libraries were constructed by using the Illumina TruSeq Stranded RNA Kit (Illumina, San Diego, CA, USA) following the manufacturer’s recommendations. The quality and quantity of the libraries were assessed with an Agilent 2100 Bioanalyzer and an ABI StepOnePlus Real-Time PCR System, respectively. Purified cDNA (20 ng/µl) from each line within the bulks was combined in equal amounts to prepare tolerant and sensitive bulk libraries. The cDNA libraries of 8 samples were sequenced on an Illumina HiSeq2500 platform strictly in accordance with standard procedures by Novogene Bioinformatics Technology Co., Ltd, China. The primary sequencing datasets generated during the current study are available Genome Sequence Archive, https://ngdc.cncb.ac.cn/gsa (accession no. CRA008748).

A total of fifteen genes from the list of possible candidate genes were selected (**Table S6**) for validation by quantitative real-time PCR (qRT–PCR) of leaf tissue. The gene sequences of the fifteen genes were downloaded from the Ensembl Plants database, and exonic sequences were used to design primers with Primer3 software (http://plants.ensembl.org/Oryza_sativa/ ). qRT–PCR with three independent biological replicates was performed using a LightCycler^®^ 96 Real-Time PCR System (Roche Life Science, Germany) and SYBR Premix without ROX based on the manufacturer’s protocol. The actin gene of rice (*Os03g0836000*) was employed as a suitable internal control gene. Transcript levels of nominated genes from three biological replicates were computed as 2^- ΔΔCt.^

#### 2.3.3 Sequence analysis of candidate gene(Os06g0184800)

To characterize the sequence variation of candidate gene(*Os06g0184800*) in RILs, a re-sequencing data analysis was performed with the gene body and the 2 kb promoter of *Os06g0184800*. PCR-based sequencing was also conducted to confirm and estimate these markers between Jileng1 and Milyang23 (Primers list on file S1). Sequence alignments of candidate genes were performed with DNAman (https://dnaman.software.informer.com/).

### 2.4 Statistics and analysis

#### 2.4.1 Genetic mapping and bin map construction

To reduce the false positive SNP genotypes in the population, consecutive SNPs were joined into one bin using a sliding window approach, and the genotype of each bin was determined based on the ratio between SNPs from the two parents (Huang et al., 2009a). The genetic linkage map was constructed based on bin markers using JoinMap4 software (Ooijen et al., 2006). In each linkage group, the maximum likelihood method was used to determine the final marker order according to the optimal AIC value, and the Kosambi mapping function was used to convert the recombination rate into genetic distance.

#### 2.4.2 Statistical analysis of SES

As the standard evaluating score data were collected from two environments and three years, best linear unbiased predictors (BLUP) were used for the overall linkage analysis by the Lme4 package in the R program. The linear model for BLUP was Y_ijk_=L_k_+E_i_+R I_ij_+(L×E)_ik_+e_ijk_, where Y_ijk_ is the observed phenotype for the k^th^ line in the j^th^ replicate of the i^th^ environment; L_k_ is the random effect of the k^th^ line; E_i_ is the random effect of the i_th_ year I(E)_ij_ is the random effect of the j^th^ replicate in the i^th^ year; (L×E)_ik_ is the random interaction effect of the i^th^ year and the k^th^ line; and e_ijk_ is the error. The heritability estimates were calculated using variance components obtained by the BLUP linear model, according to the formula (Nyquist and Baker, 1991): *H* ^2^(*%*) = $\sigma_{\text{g}}^{2}$ /( $\sigma_{\text{g}}^{2}$ + $\sigma_{\text{ge}}^{2}$ /*n* + $\sigma_{\text{e}}^{2}$ /*nr*) × 100*%* , where $\sigma_{\text{g}}^{2}$ is the genotypic variance, $\sigma_{\text{ge}}^{2}$ is the variance for interactions of genotype with environment, $\sigma_{\text{e}}^{2}$ is the error variance, n is the number of environments, and r is the number of replications. The estimated values were used as the phenotype of subsequent QTL mapping. Pearson correlation coefficients between environments and years were calculated using the PROC CORR procedure in SAS 9.4 (SAS Institute Inc, 2015) based on the BLUPs of the traits across environments.

#### 2.4.3 QTL mapping

The multi-interval mapping function (MQM) was employed to detect QTLs for standard evaluation scores based on their BLUP value using MapQTL6.0 (Van Ooijen, 2010). Locations, contribution rates and additive effects were estimated based on BLUP values of W2SES and W4SES. The LOD threshold was determined by applying 1000 permutation tests with 5% probability. The physical confidence interval of the QTL corresponded to a 2-LOD decrease from the peak LOD value, and the QTLs with overlapping candidate intervals were merged. To ascertain the stability of QTLs, we carried out QTL mapping analysis under both greenhouse and field environments. QTLs detected under all three conditions (greenhouse, field and BLUP) were considered to be stable QTLs and were used to carry out subsequent candidate gene prediction. Genes located in stable QTL intervals were annotated and analyzed based on the Rice Annotation Project (RAP) (http://rapdb.dna.affrc.go.jp ) and Ensembl (http://plants.ensembl.org/Oryza_sativa ) databases.

#### 2.4.4 Transcriptome profiling by RNA-Seq

Gene expression profiles associated with salt stress were analyzed by performing a large-scale inspection of differentially expressed genes (DEGs). The raw RNA-Seq data of eight samples (SalP1, SalP2, NorP1, NorP2, SalT, SalS, NorT and NorS) were processed. Raw data was filtered by fastp (Chen and Gu, 2018). The clean data was mapped to the Nipponbare genome (Os-Nipponbare-Reference-IRGSP 1.0, http://plants.ensembl.org/Oryza_sativa/ ), and read counts were generated using HISAT (Kim et al., 2015) with default parameters. Read counts were normalized by edgeR with TMM method (Robinson et al., 2010). Normalized read counts were used as input data for differential expression analysis in the tolerant vs. susceptible plants. Differentially expressed genes (DEGs) between Jileng1 versus Milyang 23 and tolerant versus sensitive bulks under salt stress and normal conditions were identified. The DEGs of SalT vs. SalS, NorT vs. NorS, SalP1 vs. SalP2 and NorP1 vs. NorP2 were discriminated with |log2 fold change|≥1 and a false discovery rate (FDR) ≤ 0.05.

Subsequently, a more meaningful comparison for identifying the DEGs responding to salt stress would be to examine the DEGs between (SalP1 vs. SalP2) vs. (NorP1 vs. NorP2) and (SalT vs. SalS) vs. (NorT vs. NorS). Both constitutive and stress-induced gene expression could be important for identifying salt tolerance-related DEGs. Unshared DEGs between (SalP1 vs. SalP2) and (NorP1 vs. NorP2) were both regarded as target DEGs responding to salt stress in the parents. Therefore, there were six classes of unshared DEGs that were considered: genes that were upregulIted (i, “SalOnly_up”) or downregulated (ii, “SalOnly_down”) only in the salt stress condition and unchanged in the normal condition; genes that were upregulated (iii, “NorOnly_up”) or downregulated (iv, “NorOnly_down”) only in normal conditions and unchanged in salt stress conditions; and DEGs that were detected under both salt stress and normal conditions but showed a difference in LFC (△LFC = LFC of the salt stress condition—LFC of the normal condition), (v, “△LFC > 1”) or (vi, △LFC<-1). The types i, iv and v were regarded as upregulated DEGs specifically related to salt stress, and the types ii, iii and vi were considered downregulated DEGs specifically related to salt stress (Buti et al., 2019).

Finally, such a large number of the DEGs could not be complete responsible for the variation of salt stress, as the inevitable RNA-Seq detection errors. Hence, a similar analysis process with (SalR vs. SalS) vs. (NorR vs. NorS) was also taken to explore target DEGs related to salt stress as the background noise of differential expression. Those specifically induced target DEGs were compared between (SalT vs. SalS) vs. (NorT vs. NorS) and (SalP2 vs. P1) vs. (NorP2 vs. P1), and the shared target DEGs were subjected to enrichment analyses and overlaid with stable QTL confidence intervals according to sequence and QTL location information.

## 3 Results

### 3.1 Construction of the Bin map and assessment of quality and accuracy

For proper identification of SNPs between the two parents that could be used as molecular markers, deep resequencing was performed for Jileng 1 and Milyang 23. The effective sequencing depths of Jileng 1 and Milyang 23 reached 39.2-fold and 43.8-fold, respectively. Construction of the genetic linkage map for the RILs was carried out by resequencing the 253 RILs, resulting in approximately 16,694,396 to 41,400,184, reads per line with a mean value of 24,061,543. The overall effective depth of coverage of these RILs ranged from 6.19-fold to 14.68-fold, with an average depth of 8.94-fold. The overall effective coverage (1X) of these RILs ranged from 87.49% to 98.59%, with an average coverage of 93.58%.

A total of 1,860,935 high-quality, biallelic, homozygous SNP markers were detected between ‘Jileng 1’ and ‘Milyang 23’ and used to construct a bin map (**Table 1**; **Supplementary Table S1**). Using the sliding window approach (Huang et al., 2009a), a total of 3,061 bin markers were created, and the average physical interval of the adjacent bins was 121.94 kb. Each chromosome contained 255 bins on average, among which the maximum number of bins was 366 on chromosome 1, and the minimum number of bins was 181 on chromosome 9 (**Table 1**).

**Table 1:** Characteristics of the high-density genetic map based on bin markers.

Chromosome	Bin number	Bin average length(kb)	LG Length(cM)	Average gap(cM)	Max gap(cM)
1	349(366) ^a^	118.23	165.63	0.47	2.36
2	297(304)	118.21	129.4	0.44	1.76
3	286(290)	125.56	157.38	0.55	3.02
4	260(275)	129.10	123.2	0.47	2.59
5	202(213)	140.65	116.44	0.58	2.79
6	246(261)	119.73	125.74	0.51	4.97
7	240(255)	116.46	96.62	0.40	2.91
8	230(250)	113.77	123.04	0.53	3.7
9	168(181)	127.14	79.51	0.47	4.81
10	206(215)	107.94	78.82	0.38	1.74
11	239(252)	115.16	108.19	0.45	2.66
12	198(199)	138.35	104.08	0.53	5.66
Genome	2921(3061)	121.94	1408.03	0.48	5.66

^a^ The number in bracket represents bin markers located on Chromosome, and the number out of bracket represents bin markers was included in the linkage group building.

**Figure 1:**
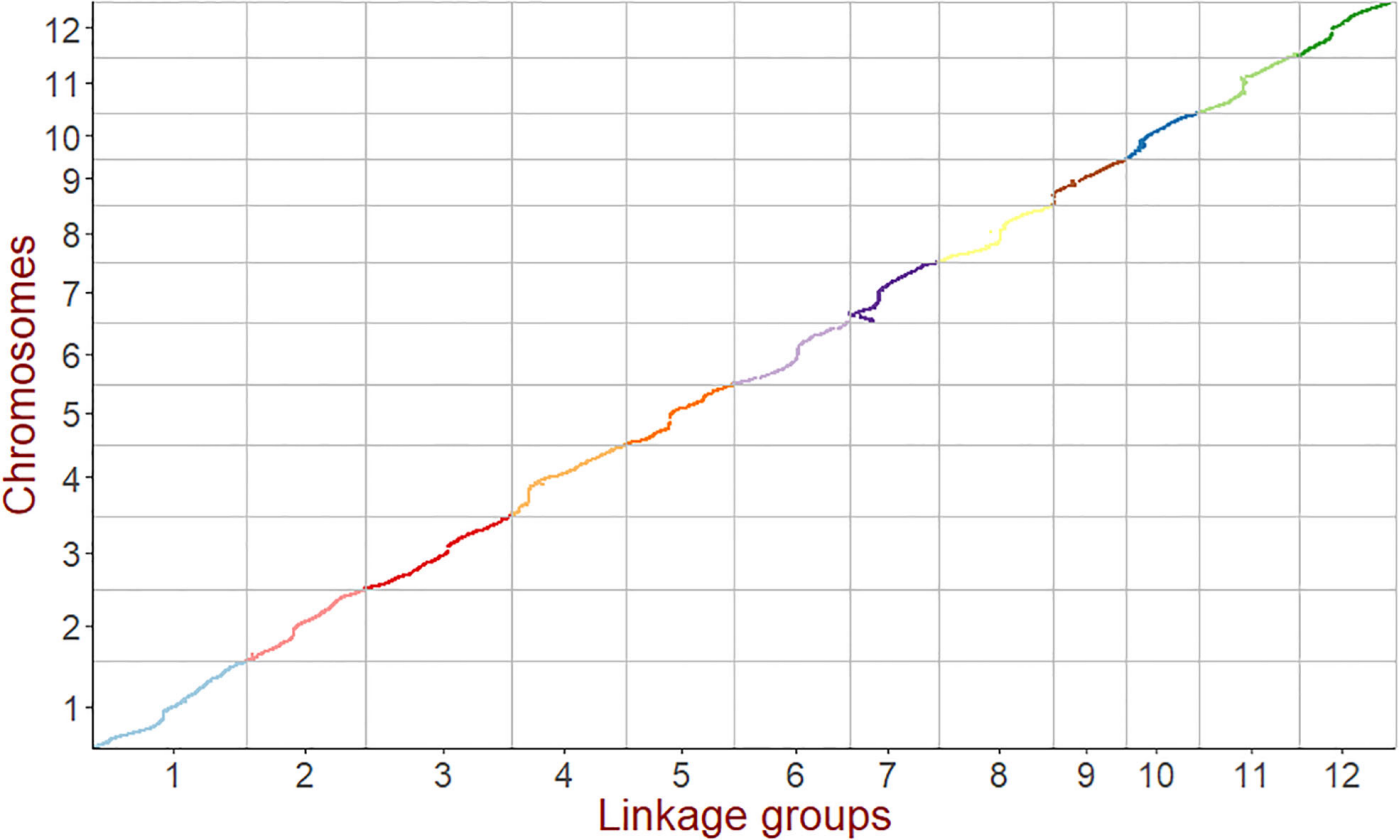
Collinearity analysis of the genetic map and genome. The x-axis is the genetic distance of each linkage group. The y-axis is the physical length of each chromosome, with the collinearity of genomic markers and genetic maps represented in the scatter plot. Different colors represent different chromosomes or linkage groups.

Bins instead of individual SNPs were used as markers to increase mapping efficiency. Finally, a high-density linkage map containing 2921 bin markers was constructed, and the total length of the genetic map was 1408.03 cM. The largest chromosome 1 had a genetic distance of 165.63 cM, and the smallest chromosome 10 had a genetic distance of 78.82 cM. All bin markers were evenly distributed across 12 chromosomes, and the average genetic distance was 0.48 cM; only one marker interval, located on chromosome 12, was larger than 5 cM **Figure 1**), and the calculation accuracy of the genetic recombination rate was high, which met the requirements of QTL mapping.

**Figure 2:**
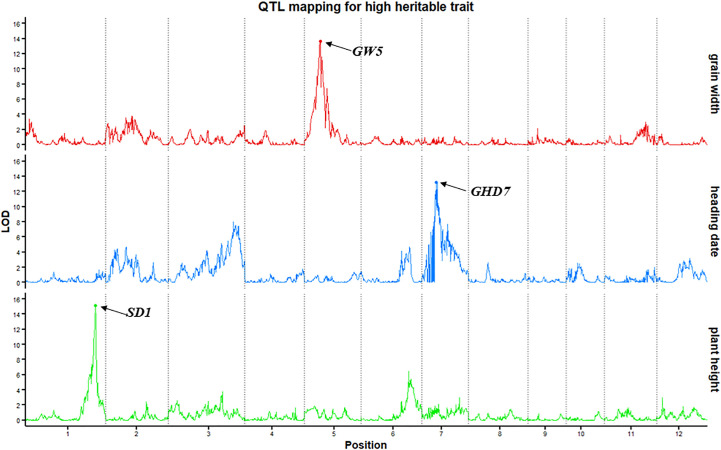
QTL mapping for highly heritable traits (plant height, heading date and grain width). The x-axis scales genetic distance on chromosomes, while the y-axis represents the LOD scores of each trait.

To evaluate the power and accuracy of this genetic map for detecting loci related to a highly heritable trait, plant height, heading date and grain width were subjected to QTL analysis in this RIL population. The QTL *qPH1-1*, whose peak encompassed the cloned gene *SD1 (Os01g0883800)* (Spielmeyer et al., 2002), was detected on chromosome 1 at position 38.41 Mb with a high LOD value of 15.16 (**Figure 2**). In addition to the *SD1* gene, QTLs detected for heading date (*qHD7-1*) (GHD7, *Os07g0261200*) (Xue et al., 2008) and grain width (*qGW5-1*) (GW5, *Os05g0187500*) (Weng et al., 2008) also verified the accuracy. Here, we detected *qHD7-1* as a major QTL (LOD=13.2) on chromosome 7 (9.22 Mb) controlling heading date and explaining 21.4% of the phenotypic variation. This QTL encompassed the cloned gene *GHD7(Os07g0261200)*. *qGW5-1* (LOD=13.76), which was located on chromosome 5 (5.43 Mb), controlled grain width, and explained 23.1% of the phenotypic variation, encompassed the cloned gene GW5 (*Os05g0187500*). Thus, mapping of the *SD1*, *GHD7* and *GW5* genes using our bin map demonstrates the accuracy of this map and shows that QTL mapping with an ultra-high-density bin map will appropriately identify genetic loci.

### 3.2 Assessing SES among the parents and RILs

Parent Jileng 1 is a salt-sensitive cultivar with a high SES (W2SES=4.5, W4SES=6.3). In contrast, the parent Milyang 23 is a salt-tolerant cultivar with a relatively low SES (W2SES=2.9, W4SES=3.7) throughout both investigation time points. The two parents showed significant difference in salt tolerance (**Table 2**).

**Table 2:** SES of salt tolerance in parents and RILs.

Trait	Parent		Mean ± SD	Range	CV(%)	Skew	Kurt.	${\text{H}}_{g}^{2}$ (%)
	Jileng 1	Milyang 23						
W2SES	4.5 ± 1.0	2.9 ± 0.6	4.3 ± 0.7	2.7-6.0	15.1	0.00	-0.36	69.5
W4SES	6.3 ± 0.8	3.7 ± 0.3	5.1 ± 0.9	2.8-7.1	16.8	-0.11	-0.13	75.5

**Figure 3:**
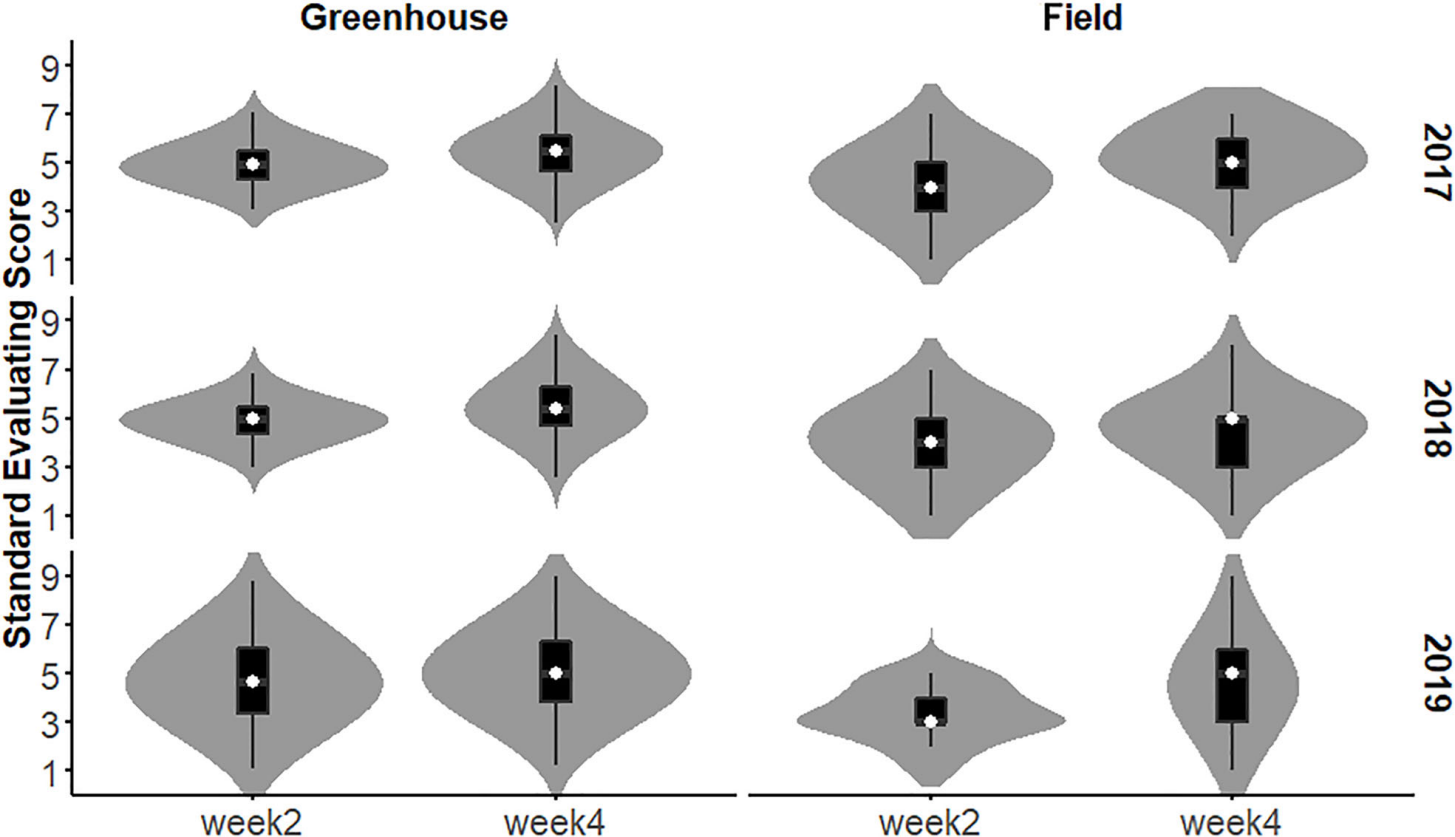
Distribution of RIL SES under greenhouse and field conditions from 2017 to 2019.

The SES of the population in each year and environment was analyzed and displayed a continuous distribution (**Figure 3**), indicating that the SES trait was controlled by multiple genes and had a quantitative inheritance pattern. We conducted ANOVA for SES across environments and years and calculated the effects of genotype (G), environment (E), and genotype-environment interactions (G×E) on the traits (**Table 2**). These traits showed high broad-sense heritability, ranging from 65.7% to 86.2%, suggesting a major role of genetic factors in the expression of these traits as well as a considerable proportion of environmental variation.

**Figure 4:**
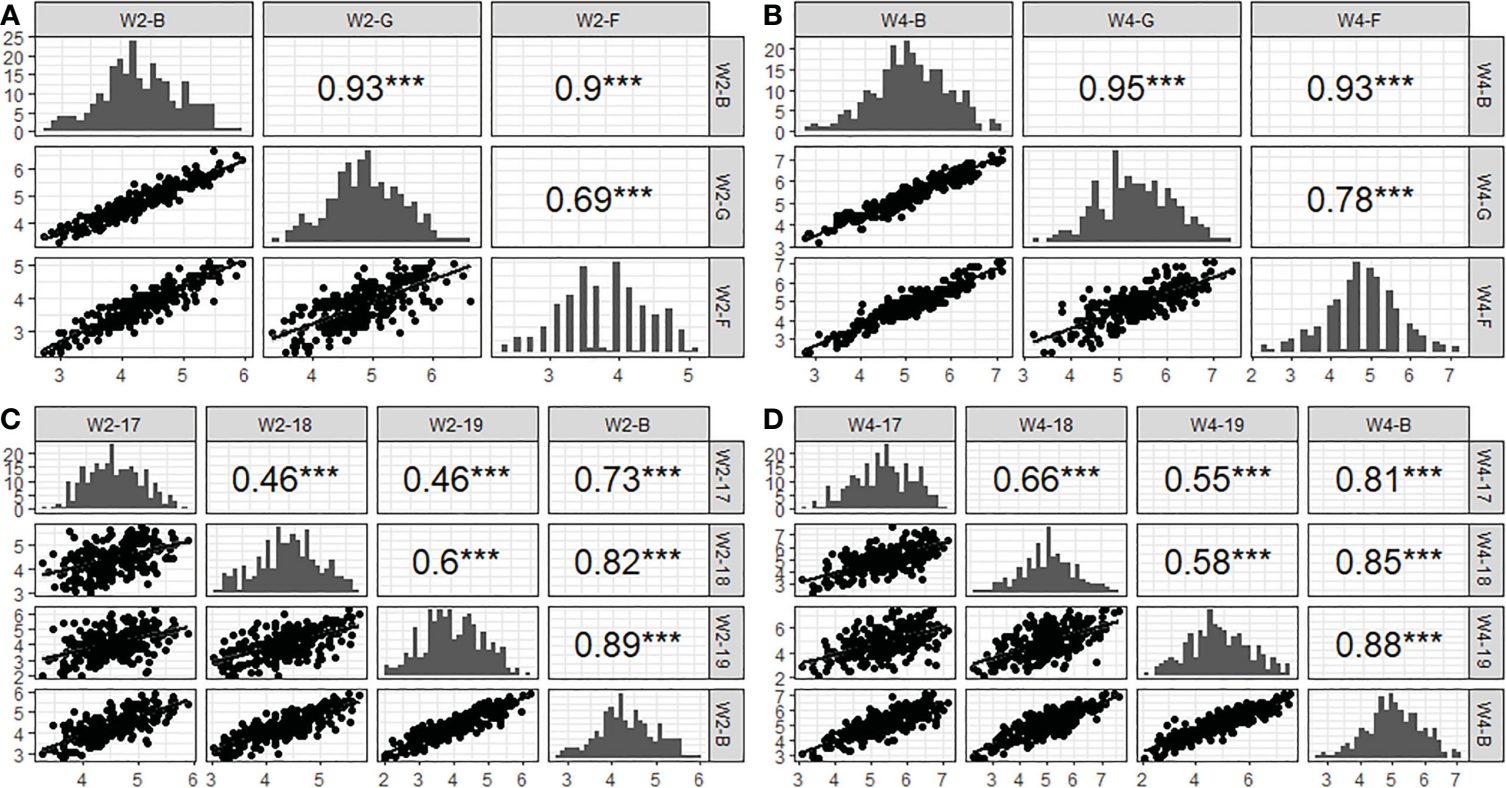
Correlation and distribution of SES from 2017 to 2019 acorss three conditions (greenhouse, field and BLUP). **(A)**: W2-B, W2-G and W2-F represent two week’ s SES of BLUP, greenhouse and field, respectively. **(B)**: W4-B, W4-G and W4-F representfour week’s SES of BLUP, greenhouse and field, respectively. **(C)**: W2-17, W2-18 and W2-19 represent two week’s SES of 2017, 2018 and 2019, respectively. **(D)**: W4-17, W4-18 and W4-19 represent four week’s SES of 2017, 2018 and 2019, respectively. *** indicated the level of significance reached 0.001.

To elucidate the repeatability of SES across populations, correlation coefficients (r) between years and environments were calculated. The results showed that the correlation coefficient of W2SES and W4SES between the field and greenhouse reached 0.69 and 0.78, respectively, which was extremely significant. From 2017 to 2019, the correlation coefficients of the SES between populations after two weeks of salt treatment ranged from 0.46 to 0.6 in different years, reaching extremely significant levels. The correlation coefficients of the SES between populations after 4 weeks of salt treatment ranged from 0.55 to 0.66 in different years, also reaching extremely significant levels. The results of the correlation analysis showed that the salt tolerance of the population was highly correlated in different environments, and the repeatability of the experiment was good (**Figure 4**).

**Figure 5:**
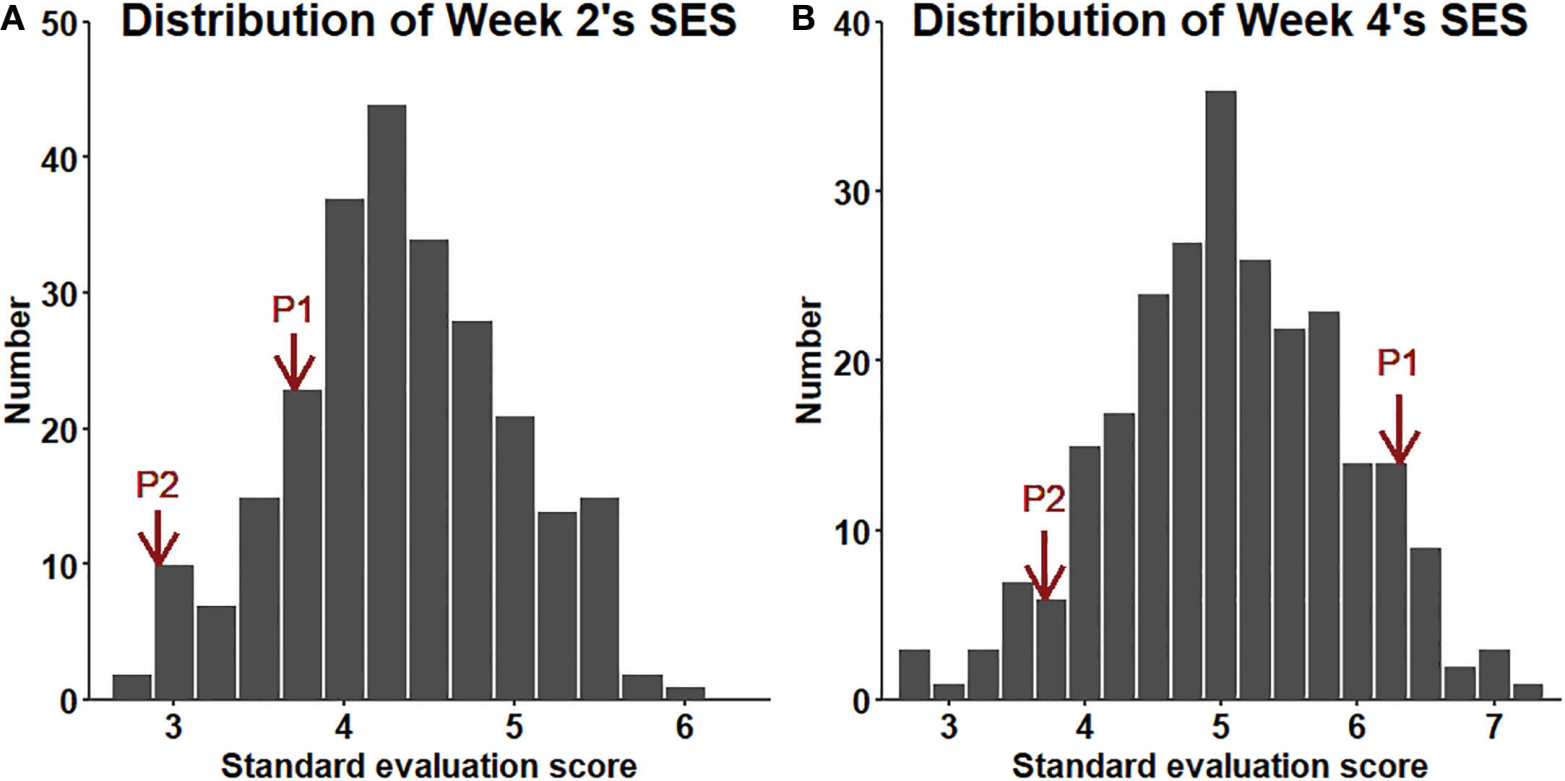
**(A)**: Distribution of the SES (BLUP) values of the RILs after two weeks of salt treatment. **(B)**: Distribution of the SES (BLUP) values of the RILs after four weeks of salt treatment.

The salt tolerance of the RILs across different environments was evaluated (**Figure 5**). The mean SES at two weeks was 4.3, with a range of 2.7-6.0 and a coefficient of variation of 15.1%. The mean SES at 4 weeks reached 5.1, with a range of 2.8-7.1 and a coefficient of variation of 16.8%. The range of the variation in both W2SES and W4SES was large, showing transgressive segregation. The skewness and kurtosis of SES were between -1 and 1, presenting a normal distribution. The generalized heritability of W2SES and W4SES reached 69.5% and 75.5%, respectively. This result indicated that the salt tolerance of the population is greatly affected by genetic factors. The best linear unbiased predictions of W2SES and W4SES were used for the subsequent QTL mapping.

### 3.3 QTL analysis

**Figure 6:**
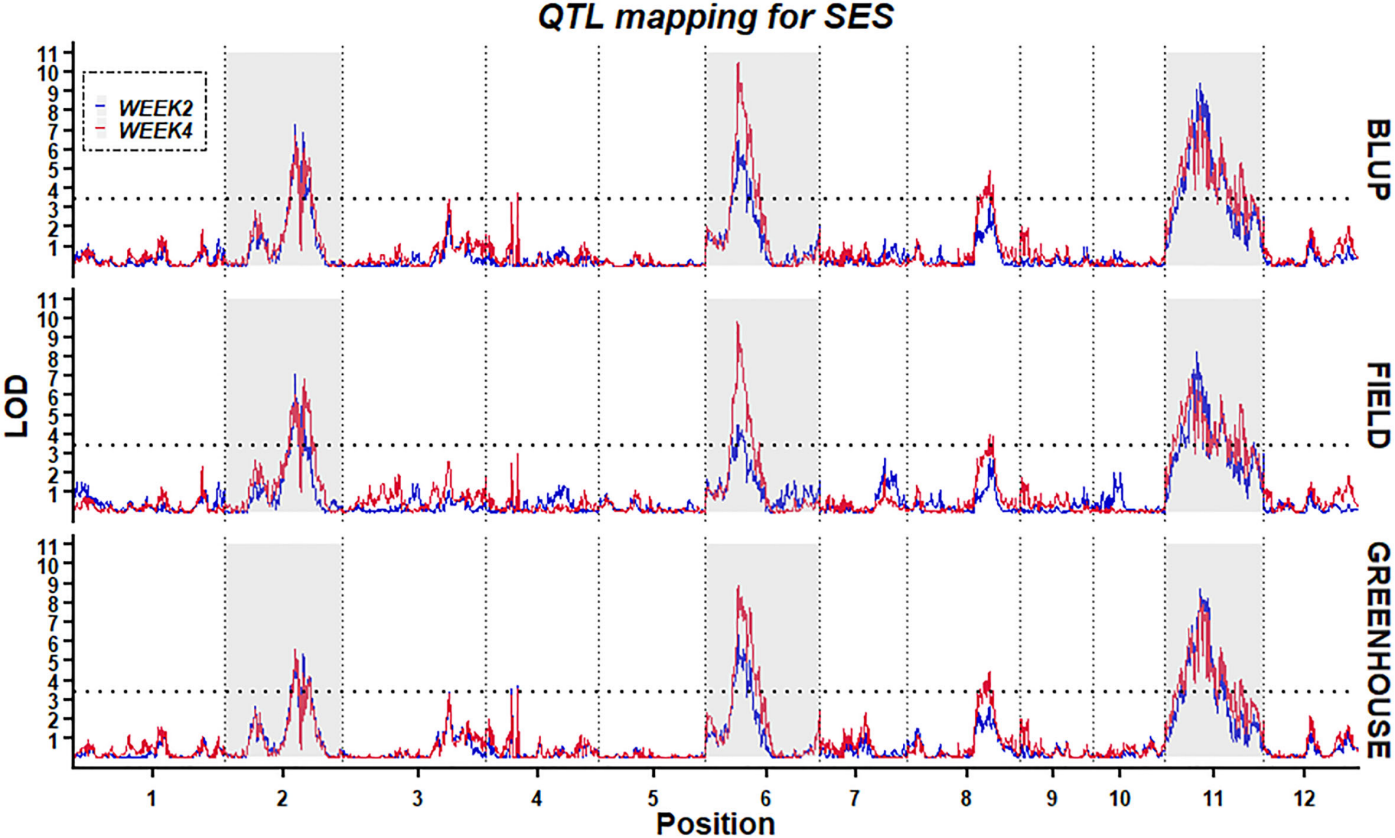
Stability of QTL mapping. The x-axis scales genetic distance on chromosomes, while the y-axis represents the LOD scores of W2SES and W4SES.

Using the high-density bin genetic map and BLUP analysis, twelve QTLs for two traits (W2SES and W4SES) across two environments were identified and explained 6.0–17.4% of the phenotypic variance (**Figure 6**; **Table 3**). All of the QTLs related to salt stress showed an additive effect contributed by Jileng 1.

**Table 3:** QTL mapping of BLUP values of W2SES and W4SES.

Trait	QTL	Chromosome	LOD	Genetic Interval(cM)	Physical Interval(Mb)	PVE(%)	Additive
W2SES (4*)	*qW2SES*2.1	2	7.32	75.68-79.81	21.94-23.11	12.5	0.24
	*qW2SES*6.1	6	6.46	34.05-44.79	4.13-5.61	11.1	0.22
	***qW2SES*11.1**	**11**	**9.43**	**36.63-40.57**	**6.65-7.63**	**15.8**	**0.26**
	*qW2SES*11.2	11	5.97	57.42-67.57	18.62-20.74	10.3	0.22
W4SES (8)	*qW4SES*2.1	2	6.68	71.95-79.81	21.15-23.11	11.5	0.29
	*qW4SES*3.1	3	3.42	112.66-118.05	27.64-28.64	6.0	0.21
	*qW4SES*4.1	4	3.77	34.42-34.81	15.81-18.24	6.6	0.23
	***qW4SES*6.1**	**6**	**10.5**	**34.33-37.32**	**4.13-4.84**	**17.4**	**0.36**
	*qW4SES*8.1	8	4.87	77.19-94.96	20.54-24.05	8.5	0.25
	*qW4SES*11.1	11	8.24	37.06-40.57	6.83-7.63	13.9	0.32
	*qW4SES*11.2	11	9.43	58.29-66.15	18.70-20.45	11.3	0.29
	*qW4SES*11.3	11	5.97	81.94-88.01	24.25-25.43	9.2	0.26

* The number in brackets represents QTL detected for corresponding trait, and the bold represents main effect QTL whose contribution reached more than 15%.

After two weeks of salt treatment, four QTLs related to SES were identified. These QTLs were located on chromosome 2 (*qW2SES2.1*), chromosome 6 (*qW2SES6.1*) and chromosome 11 (*qW2SES11.1*, *qW2SES11.2*), and all of them explained at least 10% of the variation. Among them, the QTL *qW2SES11.1*, whose contribution reached 15.8%, was identified as the major QTL for W2SES. After four weeks of salt treatment, eight QTLs related to SES were identified, and they were distributed on chromosomes 2, 3, 4, 6, 8 and 11 respectively. The variance explained by *qW4SES2.1*, *qW4SES6.1*, *qW4SES11.1* and *qW4SES11.2* was more than 10%, and those QTLs identified for W2SES in the meanwhile. Among them, the QTL *qW4SES6.1*, whose contribution reached 17.4%, was identified as the major QTL for W4SES.

To ascertain the stability of the detected QTLs, linkage analysis was individually applied to the W2SES and W4SES values for RILs under field and greenhouse environments. Five physical regions located on chromosomes 2, 6, 8 and 11 were repeatedly detected across the field, greenhouse and BLUP and could be considered environmentally stable QTLs (**Table 4**). Significantly, three stable QTLs that were located on chromosome 2: 20.92 Mb-23.44 Mb, chromosome 6: 4.13 Mb-4.85 Mb, and chromosome 11: 6.65 Mb-8.34 Mb were detected across all environments and evaluation time. Thus, given their value and reliability, these QTLs should be strongly considered for exploring candidate genes.

**Table 4:** Comparing QTL confidence interval among greenhouse, field and BULP.

Chr.	Genetic Interval (cM)	Physical Interval(Mb)	Greenhouse		Field		BLUP	
			W2SES	W4SES	W2SES	W4SES	W2SES	W4SES
2	70.60-80.44	20.92-23.44	√	√	√	√	√	√
6	31.63-45.10	4.13-4.85	√	√	√	√	√	√
8	75.65-96.15	19.81-24.23		√		√		√
11	36.36-42.05	6.65-8.34	√	√	√	√	√	√
11	80.03-88.01	23.65-25.43		√		√		√

### 3.4 Gene expression profile and comparative transcriptome analysis

To complement the QTL mapping and help identify salt stress-responsive genes, the extent of transcriptomic differences in response to salt stresses between the two parents of the RILs was determined by RNA-Seq. As shown in **Table 5**, under salt stress, compared to Jileng 1, 2081 upregulated and 3002 downregulated DEGs were identified in Milyang 23. Even without salt stress, there were 1603 constitutively overexpressed and 2934 constitutively underexpressed DEGs between the two parents. To remove the background noise caused by constitutive variation between the parents, those DEGs detected under normal conditions were taken as the baseline control. By comparing and removing these baseline DEGs, we retained 1484 SalOnly_up, 1840 SalOnly_down, 1006 NorOnly_up, 1772 NorOnly_down, 265 “△LFC>1”, and 498 “△LFC<-1” DEGs that were specific to salt stress. The types SalOnly_up, NorOnly_down and △LFC>1 were regarded as upregulated DEGs specifically related to salt stress, and the types SalOnly_down, NorOnly_up and △LFC<-1 were considered downregulated DEGs specifically related to salt stress. As a result, there were a total of 6,865 salt stress-specific DEGs, with 3,521 upregulated and 3344 downregulated DEGs.

**Table 5:** Number of genes which were differentially regulated between susceptible or tolerant genotypes in response to satt stress, according to their classification in the six classes.

Regulated to Salt-stress	DEGs Type	P1 versus P2	Sensitive versus tolerant Bulk	Common
Up	SalOnly_up(i)	1484	1040	248
	NorOnly_down(iv)	1772	449	
	△LFC>1(v)	265	8	
Down	SalOnly_down(ii)	1840	1666	303
	NorOnly_up(iii)	1006	438	
	△LFC<-1(vi)	498	17	
Total		6865	3618	551

Not all of the DEGs are likely to be responsible for the variation in salt stress, as detection errors are inevitable. Therefore, the difference in the transcriptomic profiles between salt-tolerant and salt-sensitive bulks were analyzed. As a result (**Table 5**), 1,482 upregulated and 2,135 downregulated salt stress-specific DEGs were detected between the bulks. The identified salt stress-specific DEGs between the parents and bulks were combined to decrease the background noise, and the number of salt stress-specific DEGs was drastically reduced to only 551. Those overlapping DEGs were taken as potential candidates for salt tolerance genes.

### 3.5 Colocalization of differentially expressed genes in QTL regions

Subsequently, those salt stress-specific DEGs were overlaid on the stable QTL confidence intervals to reduce the number of candidate genes. A total of 551 salt stress-specific DEGs were compared with the candidate genes residing in the stable QTLs. Markers flanking the stable QTLs were used to anchor them to the Japonica Nipponbare reference genome (IRGSP-1.0), and 989 annotated genes were obtained from five stable QTLs. Based on the combined, fifteen highly promising candidate genes were identified. The functional annotations of those candidates are listed in **Table 6**. Of these candidates, two were located on chromosome 2: 20.92 Mb-23.44 Mb, two were located on chromosome 6: 4.13 Mb-4.85 Mb, nine were located on chromosome 8: 19.81Mb-24.23 Mb, two were located on chromosome 11: 6.65 Mb-8.34 Mb and no candidates located on chromosome 11: 23.65 Mb-25.43 Mb.

**Table 6:** The promising genes associated with salt tolerance.

GeneID	Position	QTL	Gene name/function
*Os02g0568600*	chr02: 21625022.21625725	*qW2SES2.1/ qW4SES2.1*	Zinc finger protein, putative, expressed.
*Os02g0570400*	chr02: 21765011.21773418	*qW2SES2.1/ qW4SES2.1*	*OsDTC1*, Similar to Ent-kaurene synthase 1A.
*Os06g0184800*	chr06: 4264424.4265115	*qW2SES6.1/ qW4SES6.1*	*OsRCI2-8*, similar to Low-temperature induced protein lt101.1(Blt101.1).
*Os06g0184900*	chr06: 4269116.4270614	*qW2SES6.1/qW4SES6.1*	*OsHCT3*, Hydroxycinnamoyl transferase 3, Transferase family protein.
*Os08g0414700*	chr08: 19830425.19836080	*qW4SES8.1*	Similar to Trehalose-6-phosphate synthase (Fragment).
*Os08g0417000*	chr08: 19950692.19952388	*qW4SES8.1*	Oxidoreductase, 2OG-FeII oxygenase domain containing protein, putative, expressed
*Os08g0417100*	chr08: 19954825.19956227	*qW4SES8.1*	Oxidoreductase, 2OG-FeII oxygenase domain containing protein, putative, expressed
*Os08g0434300*	chr08: 21054660.21056008	*qW4SES8.1*	lactate/malate dehydrogenase, putative, expressed
*Os08g0440800*	chr08: 21445787.21450773	*qW4SES8.1*	*OsALDH11A3*, Glyceraldehyde-3-phosphate dehydrogenase.
*Os08g0450700*	chr08: 22003703.22006379	*qW4SES8.1*	Chaperonin Cpn60/TCP-1 family protein.
*Os08g0458200*	chr08: 22462399.22464860	*qW4SES8.1*	Oxidative Stress 3-like 6, Similar to MTD1
*Os08g0474866*	chr08: 23403049.23404168	*qW4SES8.1*	Similar to gibberellin receptor GID1L2
*Os08g0480000*	chr08: 23712632.23717520	*qW4SES8.1*	Multi antimicrobial extrusion protein. MatE family protein
*Os11g0234200*	chr11: 7125124.7128857	*qW2SES11.1/qW4SES11.1*	Zinc finger RING/FYVE/PHD-type domain containing protein, putative, expressed
*Os11g0235700*	chr11: 7221675.7228567	*qW2SES11.1/qW4SES11.1*	Similar to *FHY1*

Both those located in stable QTL regions and those detected by comparative transcriptome analysis were assumed to be promising candidate genes in this study.

**Figure 7:**
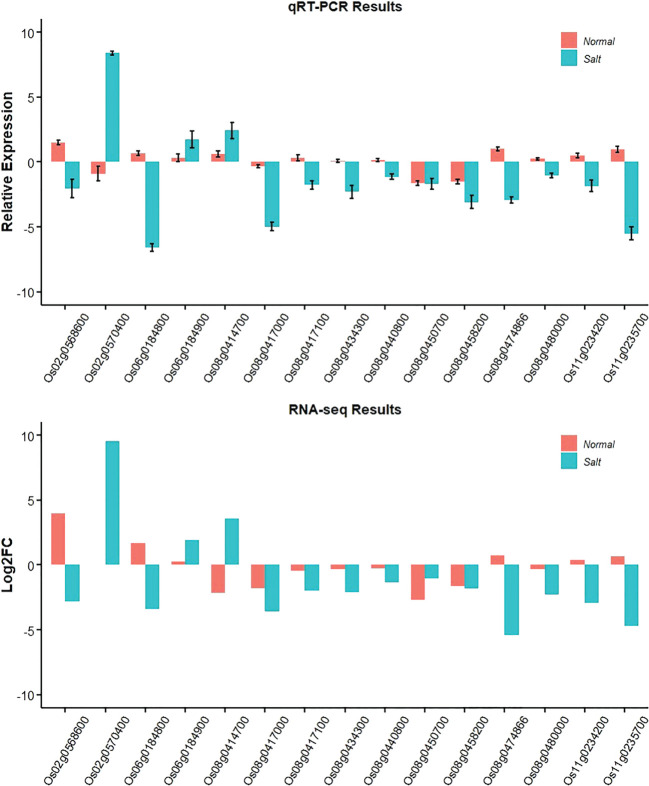
Gene expression changes under salt stress and normal conditions based on qRT-PCR and RNA-Seq.

To confirm the accuracy and reproducibility of the Illumina RNA-Seq results, those promising genes were compared for their expression level differences between Jileng 1 and Milyang 23 by quantitative real-time PCR (qRT–PCR) analysis under normal and salt stress conditions. The validation results for the fifteen genes are shown in **Figure 7**. The gene expression trend was basically consistent with the RNA-Seq analysis, supporting the reliability of the data from RNA sequencing. Functional annotation of the genes in stable QTL regions helped to reveal the biological functions of those genes. The functional annotation and gene expression data suggested that *Os06g0184800* might be the most a plausible candidate for *qW2SES6.1*/*qW4SES6.1*, but genetic modification complementation or gene editing verification is needed. *Os06g0184800* are highly promising functional candidate genes for salt tolerance.

**Figure 8:**
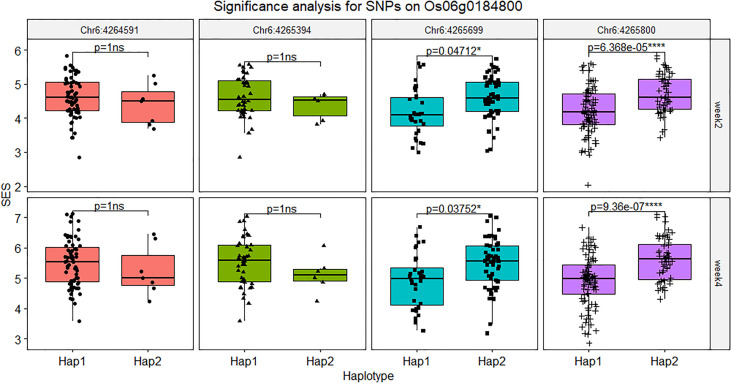
Significance analysis for SNPs on *Os06g0184800*. The symbol “ns” indicated not reach significant difference, * indicated the level of significance reached 0.05, **** indicated the level of significance reached 0.0001.

The SNPs located on the gene body and the 2 kb promoter of Os06g0184800 were screened out and fifity-four SNPs were found in this region and five SNPs were verified by PCR-based sequencing between Jileng1 and Milyang23, which four SNPs located on promoter and one SNP located on the gene body (**Table S10**). In the gene body, the SNP (Chr6: 4264591, C to T), was detected on the 3’ UTR region with C to A transition. In the promoter, the four SNPs were respectively detected on position -279 (Chr6: 4265394, C to T), -526 (Chr6: 4,265,630, insert A), -585 (Chr6:4265699, G to A), -766 (Chr6: 4,265,880, T to C) from transcription start site (ATG) of *Os06g0184800* (**Figure S4**). Based on the significance test on haplotype of those markers, except Chr6: 4265630 with too much missing from resequence, Chr6:4265699 and Chr6: 4265880 shown extremely significant difference bewteen their haplotype, and Chr6: 4265394 and Chr6: 4265630 didn’t show difference (**Figure 8**).

## 4 Discussion

### 4.1 High-density bin genetic mapping assisted in precise QTL exploration

Linkage analysis is an efficient approach to understanding the genetic basis of quantitative traits in crops (Bohra et al., 2020). Genetic map was fundamental to precise linkage analysis (Xie et al., 2010). Parental genetic diversity and marker density are the major factors affecting the efficiency and accuracy of QTL mapping. Previously, the insufficiency of molecular markers were major limitation in discovering QTLs in rice (Bohar et al., 2020). Many QTL studies have been performed using sparse genetic maps constructed using RFLP and SSR markers, resulting in large intermarker intervals (Lander and Botstein, 1989; Lin et al., 1998; Koyama et al., 2001; Chai et al., 2014).

Generally, increasing marker density is an effective means of improving QTL mapping resolution (Bevan et al., 2017). The rapid development of high-throughput next-generation sequencing genotyping platforms has provided the extensive capacity to develop comprehensive polymorphic markers , including SNPs and InDels.The use of whole genome resequencing and the construction of bin maps has been shown to improve efficiency of QTL mapping and accelerate genes discovery in rice (Huang et al., 2009a; Xie et al., 2010). In the current study, 2921 bin markers developed from 1,860,935 high-quality, biallelic, homozygous SNPs were used to construct a high-density genetic map covering 1,408.03 cM, with an average interval of 0.48 cM between adjacent bin markers (**Table 1**), which significantly increased the QTL mapping resolution compared with traditional markers. Compared to the average interval of 9.00 cM between adjacent markers in the previous SSR/InDel/single SNP map also derived from Jileng 1×Milyang 23 (Zhang et al., 2021), the marker density of this genetic map was observably increased (7.83 markers/Mb vs 0.78 markers/Mb), and the confidence interval of QTLs was significantly reduced.

The quality and accuracy of this genetic map was assessed by identifying known genes underlying QTL mapping from other studies. The precision and accuracy of linkage analysis were significantly improved based on the high-density bin genetic map, and was sufficient to accurately map *SD1* (Spielmeyer et al., 2002), *GHD7* (Xue et al., 2008), and *GW5* (Weng et al., 2008), which control plant height, heading date and grain width, respectively (**Figure 1**). For example, basing on this study’s high-density genetic map of Jileng1×Milyang23 RILs, *qPH1-1*, which controls plant height, was located on the interval Chr01:38,008,469bp - 38,642,574bp (634.11 kb) and explained 24.1% of the phenotypic variance, including the famous green revolution gene *SD1*. Taking the previous linkage analysis with the same RILs for comparison, a major QTL for plant height had been located in the interval Chr01:34, 949, 898 bp-36, 728, 487 bp (1.78 Mb) and explained 10.30–17.99% of the phenotypic variance (Tang, 2019). This QTL interval of current study narrowed significantly compared to previous studies and it was in more close proximity to functional gene *SD1*.

Combining this genetic map and precise phenotype, this population is competent for identifying major QTL and functional gene.

Gene prediction, however, depended also on the high accuracy and collinearity of the region of interest between genetic map and reference genome. In current study, a high collinearity between genetic map and Nipponbare reference genome (IRGSP-1.0) (**Figure 1**) indicated that this map was suitable for subsequent gene discovery.

### 4.2 Environmentally stable and major QTLs for salt tolerance

Salt tolerance is quantitative trait controlled by multiple genes with a complex genetic mechanism (Horie et al., 2012). To date, the rapid development of next-generation sequencing has accelerated QTL mapping and gene discovery in crops, and more than one thousand rice salt-tolerance QTLs have been identified (http://gramene.org/ ; Jing and Zhang, 2017). Despite many attempts using different strategies to discover functional genes controlling salt tolerance in rice, the achievements thus far have been quite modest. Only rare genes with significant effects have been thoroughly studied and cloned (Ganie et al., 2021). The inefficient exploration of salt stress-related genes is partial due to that those QTLs were mainly detected by low-density markers with large confidence intervals that contained too many genes and lacked reliability and stability. In the current study, an ultra-high-density genetic map which could rapidly anchor major genes was utilized to improve the precision of QTL positioning and narrow the range of candidate genes. Moreover, the SES values of populations across two environments and three years were evaluated to assure the accuracy of the phenotype. Through the above process, five stable QTLs were discovered.

Among the five stable QTLs, two major QTLs (*qW2SES11.1* and *qW4SES6.1* explained more than 15% phenotypic variance) were worth to lucubrate.

### 4.3 Validation of QTLs across mapping populations

Validation of QTLs in different genetic backgrounds/environments is required before they can be used in marker-assisted selection or gene cloning to rule out statistical errors (Price, 2006). Among the QTLs identified in this study, four stable QTLs partially or completely overlapped with the chromosomal regions carrying loci detected by previous studies.

The QTL *qSSR2.3*, published by Xu et al. (2020), controlling seedling survival under salt stress, was colocated on a region of chromosome 2 (71.95 cM-79.81 cM, 21.15 Mb-23.11 Mb) that contained *qW2SES2.1* and *qW4SES2.1*. Chai et al. (2014) discovered two QTLs (*qGW6.2* and *qGYP6.2*) affecting 1000 grain weight and yield per plant under salt stress, and these QTLs overlapped with a region on chromosome 6 (34.05 cM-44.79 cM, 4.13 Mb) containing *qW2SES6.1* and *qW4SES6.1*. On chromosome 11 (36.63 cM – 40.57 cM, 6.65 Mb-7.63 Mb), Chai et al. (2014) also identified two QTLs (*qSF11.5* and *qGYP11.7*) affecting seed setting rate and yield per plant under salt stress, and Puram et al. (2017) located two QTLs (*qSHL11.1* and *qRHL11.1*) for bud length and root length under salt stress. On chromosome 11 (57.42 cM -67.57 cM, 24.25-25.43 Mb), Xu et al. (2020) found a QTL for seedling survival time under salt stress (*qSSD11.1*), and Shen et al. (2009) found a QTL for chlorophyll fluorescence under salt stress (*qSFM-11*). The mutual comparative result reflect the accuracy of this study, as well as indicated that those QTL regions are also consistent across more than one genetic background. Thus, it was reasonably surmise the existence of salt tolerance-related genes in those intervals. These results not only strengthen the findings of previous studies but also reflect the complexity of salt tolerance in accelerating the breeding programs for enhanced salt-tolerance among rice cultivars.

There are many similarities in the genetic mechanisms controlling plant resistance to various abiotic stresses. Drought- and cold tolerance-related genes in rice can also play an important role in salt tolerance, which was further confirmed by the results of the current study. Han et al. (2007) identified QTLs affecting cold tolerance at the seedling stage (*qCSH2*, Chr2:21.66 Mb-26.76 Mb), employing the F_2:3_ population constructed by the same parents of this RIL population. Hemamalini et al. (2000) located a QTL controlling root density under drought stress (*qRTT2-1*, Chr2:20.72 Mb-25.43 Mb) that partially overlapped with the QTL interval we identified on chromosome 2 (71.95 cM-79.81 cM, 21.15 Mb-23.11 Mb). Li et al. (2005) found a QTL (*qRRSD6b*, Chr6:4.84 Mb-5.43 Mb) that controlled the ratio of root dry weight to root fresh weight under drought stress, and Dai et al. (2004) found a QTL (*qRCT6b*, Chr6:4.93 Mb-5.43 Mb) that affected the cold tolerance of rice at the reproductive stage; both of which partially overlapped with the QTL interval of chromosome 6 (34.05 cM-44.79 cM, 4.13 Mb-5.61 Mb) identified from the current study. Toojinda et al. (2003) mapped SSR markers affecting rice flooding tolerance (RM206, 24.32 Mb) to a QTL interval on chromosome 11 (81.94 cM-88.01 cM, 24.25 Mb-25.43 Mb). The results of this study further verified that within these QTL confidence intervals, pleiotropic genes may affect multiple abiotic stresses.

### 4.4 Gene expression profile and identification of candidate genes associated with salt stress

The advantageous feature of our study was the integration of high-density QTL mapping and bulked segregant RNA-Seq analysis. High marker density is preferable for improving the resolution in QTL position and assisting in determining functional causative variations in genes (Liu et al., 2014). However, there were still 989 annotated genes underlying the five stable QTLs, making it difficult to pinpoint the genes responsible for the functional differences in salt tolerance. Obtaining gene expression information by combining bulked segregant analysis and RNA-Seq is an effective strategy for filtering out background noise and can reduce the number of DEGs (Zou et al., 2016). Transcriptome profiling of bulked RILs normalized the irrelevant DEGs between the tolerant and sensitive parents while retaining the DEGs relevant for salt tolerance or sensitivity. In current study, the transcriptome profiles of two parents and two bulks under two conditions were depicted by RNA-Seq. Through the comparative analysis, a total of 551 salt stress-specific DEGs were obtained. However, there were still too many DEGs to initiate any downstream validation studies. Integrating of high-density QTL mapping and bulked segregant RNA-Seq analysis, the number of overlaped candidate genes was dramatically reduced, and only fifteen candidate genes were obtained.

Among the fifteen highly promising candidate genes, *Os06g0184800* and *Os06g0184900* located on *qW4SES6.1*, *Os11g0234200* and *Os11g0235700* located on *qW2SES11.1* respectively, which explained more than 15% phenotypic variance and play a more important role on salt tolerance, might be the most plausible prospect for target genes. Among them, *Os06g0184800* encodes small hydrophobic polypeptides, *Os06g0184900* encodes hydroxycinnamoyl transferases (Fang et al., 2022), *Os11g0234200* encodes FYVE/PHD-type domain containing protein (Wywial and Singh, 2010), and *Os11g0235700* is similar to far-red elongated hypocoty 1 (Chen et al., 2012).

Basing on those annotation, *Os06g0184800* is highly promising and worthy of further study to determine its role in the response to salt stress. *Os06g0184800*, also called *OsRCI2-8*, which belongs to the RCI2 family, is also known as plasma membrane protein 3 (PMP3). As reported previously, this gene family encodes small hydrophobic polypeptides in maintenance of ion homeostasis, and it is responsible for salt, drought, cold, and abscisic acid (Medina et al., 2007). The homologous gene of *Os06g0184800* has been described both in many cereal crops (rice, arabidopsis maize, wheat, barley) (Mitsuya et al., 2006; Nylander et al., 2001; Mustafa et al., 2005; Fu et al., 2012) and many halophytes (sheep grass, alkali grass, plaintan) (Zhang et al., 2008), and had been verified to regulates cellular Na^+^ and K^+^ accumulation, promote seed germination and lateral root growth under abiotic stress. It is reasonable to speculated that *Os06g0184800* was the target gene for *qW2SES6.1/qW4SES6.1*, which was supported by DEGs and previous studies.

Basing on the gene expression analysis of RNA-seq and qRT-PCR, *Os06g0184800* shown significant downward adjustment under salt stress with Milyang23 vs Jileng1. So it is inferred this lower expression level may be associated with salt stress. From the subsequent haplotype analysis, two SNPs (Chr6:4265699 and Chr6:4265880) located on pro moter region of *Os06g0184800* showed significant difference in different haplotypes, which were also confirmed by the database ricevarmap2 (http://ricevarmap.ncpgr.cn/ ). Those SNPs might be suitable for the development of functional markers for salt tolerance in rice to facilitate marker-assisted breeding.

In summary, the integration of QTL mapping, RNA-Seq analysis, and gene annotation provided credible candidate genes for the identified QTLs, constitutes an effective strategy for identifying promising candidate genes involved in salt tolerance for QTL mapping, which was very important for subsequent gene function verification. The function of promising candidate genes will be validated by genetic modification complementation or gene editing verification.

## 5 Conclusion

Using the whole genome resequencing approach, a genetic linkage map with an average distance of 0.48 cM between adjacent markers was constructed based on an RIL population in rice. Comparative analysis of QTLs and fine mapping suggest the high efficiency and accuracy of this genetic map. Global mapping of QTLs affecting salt tolerance was performed, and a total of twelve loci were detected across three conditions. Including the three loci that overlapped with previous reports, a total of five were defined as stable loci. The stable QTLs were highlighted and analyzed. The results of the transcriptome analysis revealed a gene expression profile responsible for responding to salt stress, and several important differentially expressed genes were colocalized in the salt stress-related QTL regions on chromosomes 2, 6, 8 and 11. Critical loci were investigated and identified as candidate genes; these were considered suitable for functional validation and breeding utilization. This study will not only help to better understand the genes and mechanisms of salt tolerance in rice but also provide new insights that will assist in the development of breeding strategies for salt-tolerant rice.

## Data availability statement

The data presented in the study are deposited in the National Genomics Data Center (NGDC), part of the China National Center for Bioinformation (CNCB), accession number CRA008901 and CRA008748.

## Author contributions

LG and LH designed the project and LG performed all the experiments and wrote the manuscript. WZ, DC, XM, and BH assisted in conducting experiments and data analysis. QZ and LH provided the direction for the study and the correction of the manuscript. All authors contributed to the article and approved the submitted version.

## Supplementary material

Supplementary Table 1 Plant height, heading date and grain width phenotypic variation of RIL.

Supplementary Table 2 The RILs arranged by an α-lattice design (Taking the trial in greenhouse of 2018 for example)

Supplementary Table 3 Phenotypic variation of parents and RILs.

Supplementary Table 4 Population sequencing and mapping

Supplementary Table 5 Overview of SNP number

Supplementary Table 6 Information of Bin marker.

Supplementary Table 7 Information of genetic map

Supplementary Table 8 Genes regulated to salt-stress by RNA-seq analysis

Supplementary Table 9 Primer of candidate genes for qRT-PCR

Supplementary Table 10 Haplotype of SNPs on *Os06g0184800* and respective SES.

Supplementary Figure 1 Performance of parents (Jileng 1 and Milyang 23) after salt treatment and control condition

Supplementary Figure 2 Performance of RILs under two environments (greenhouse and field)

Supplementary Figure 3 The evaluation criterion of SES.

Supplementary Figure 4 Alignment of reference, Jileng 1 and Milyang23 for *Os06g0184800*
