# Supplementary figures and images for: Integrating linkage mapping and comparative transcriptome analysis for discovering candidate genes associated with salt tolerance in rice
Source: Front Plant Sci. 2023 Jan 24;14:1065334. doi: 10.3389/fpls.2023.1065334 (PMC9904508; doi:10.3389/fpls.2023.1065334)

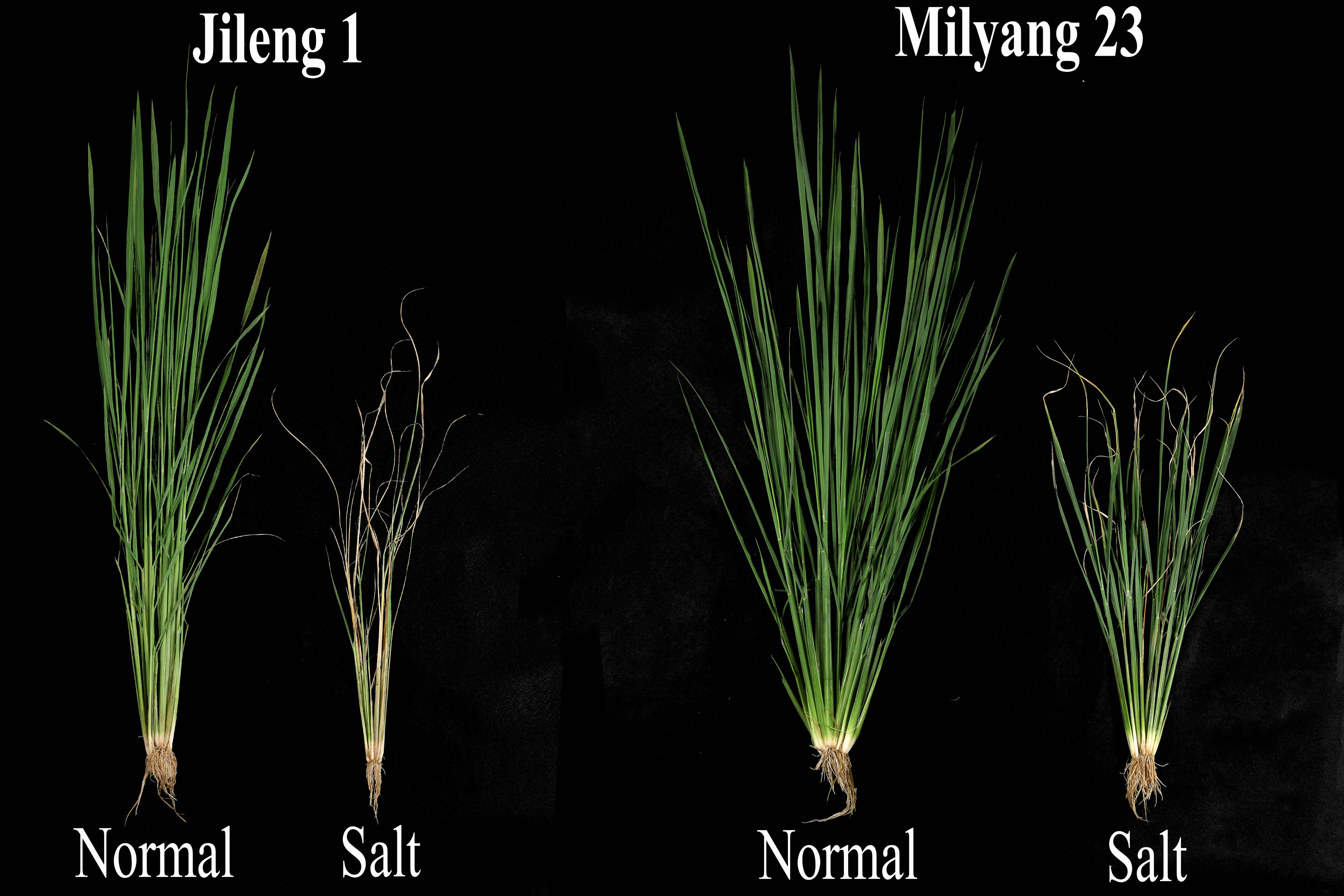

Supplement: Supplementary Figure 1 — Performance of parents (Jileng 1 and Milyang 23) after salt treatment and control condition [file Image_1.jpeg]

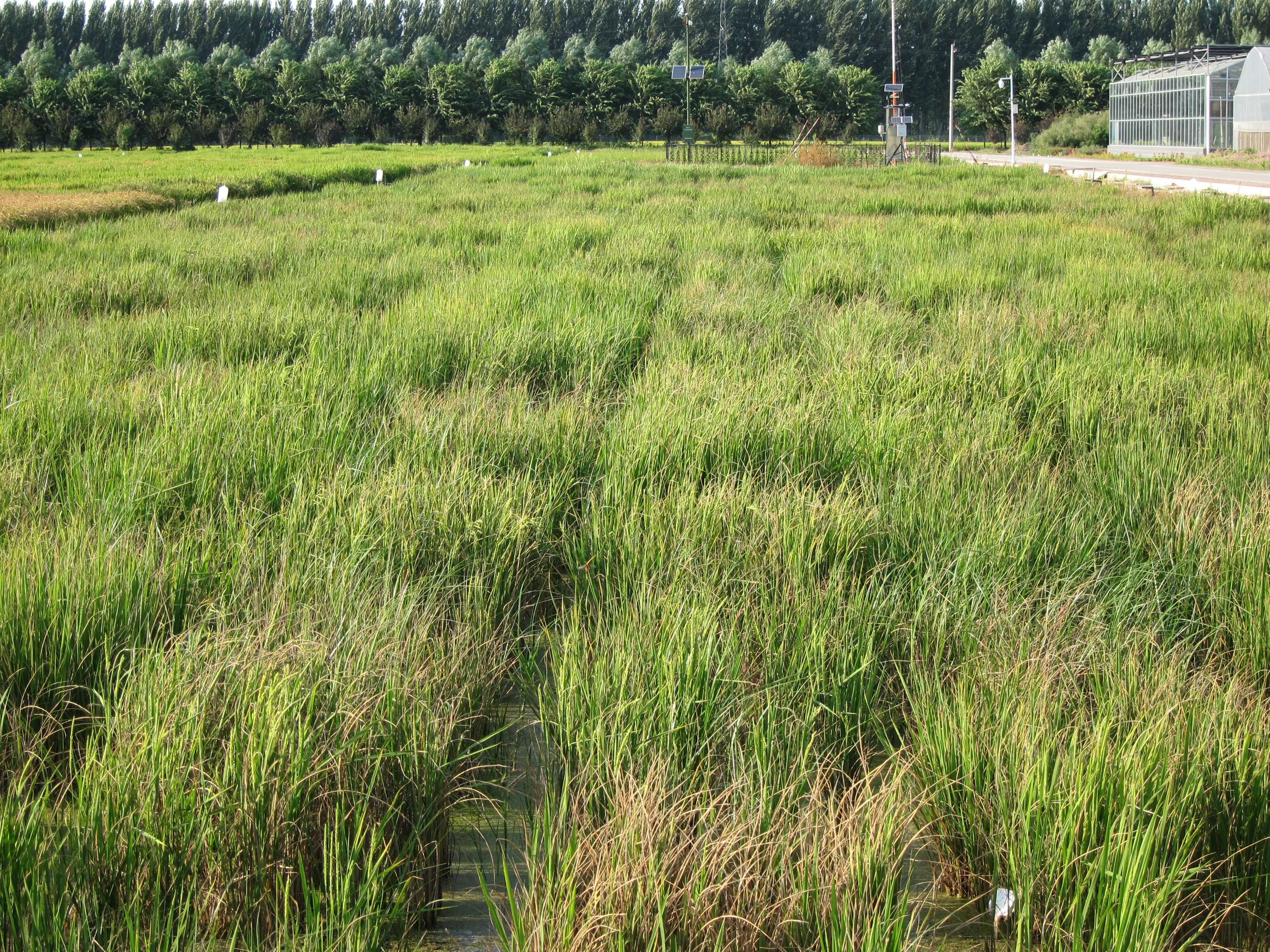

Supplement: Supplementary Figure 2 — Performance of RILs under two environments (greenhouse and field) [file Image_2.jpeg]

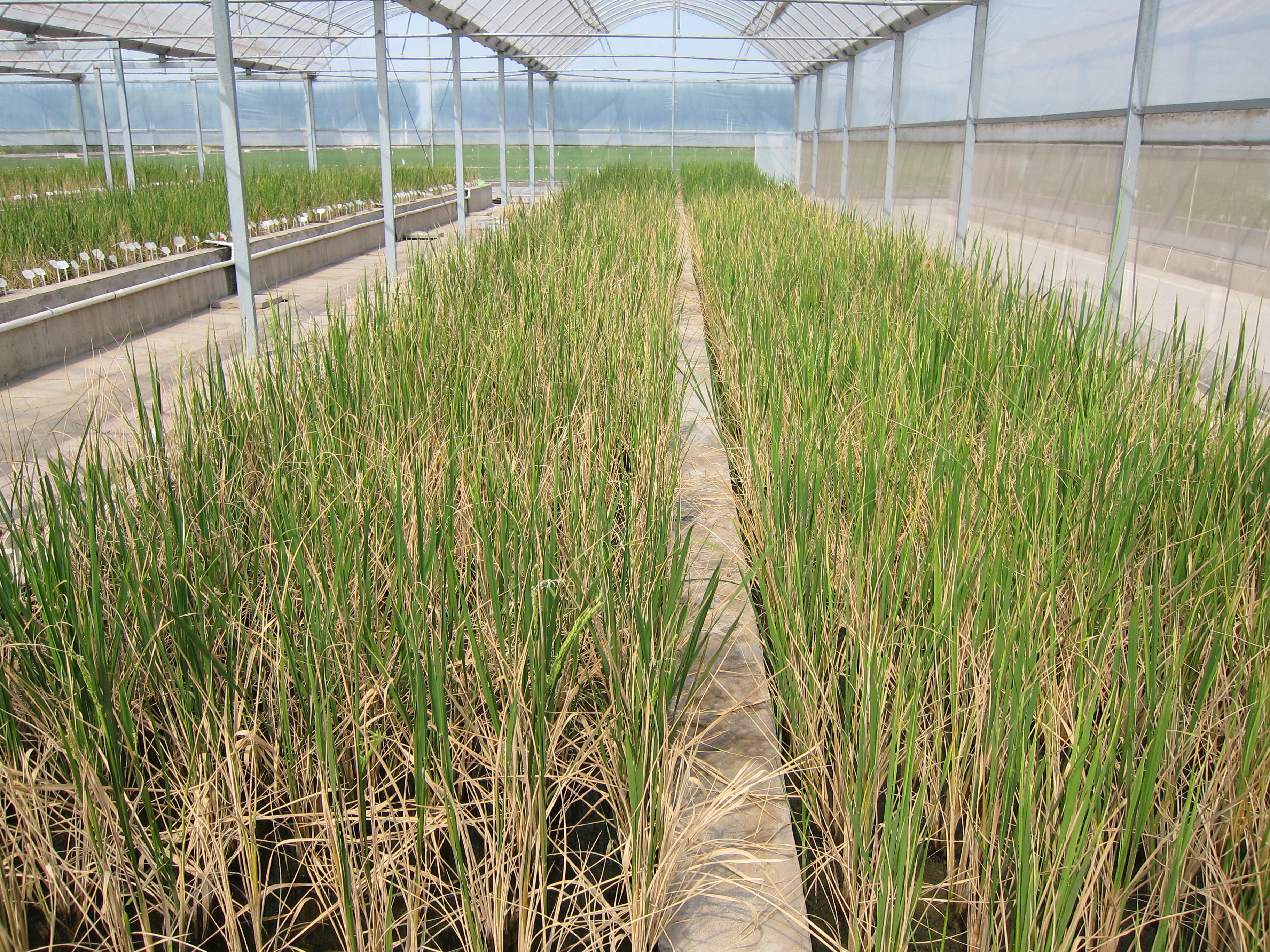

Supplement: Supplementary Figure 3 — The evaluation criterion of SES. [file Image_3.jpeg]

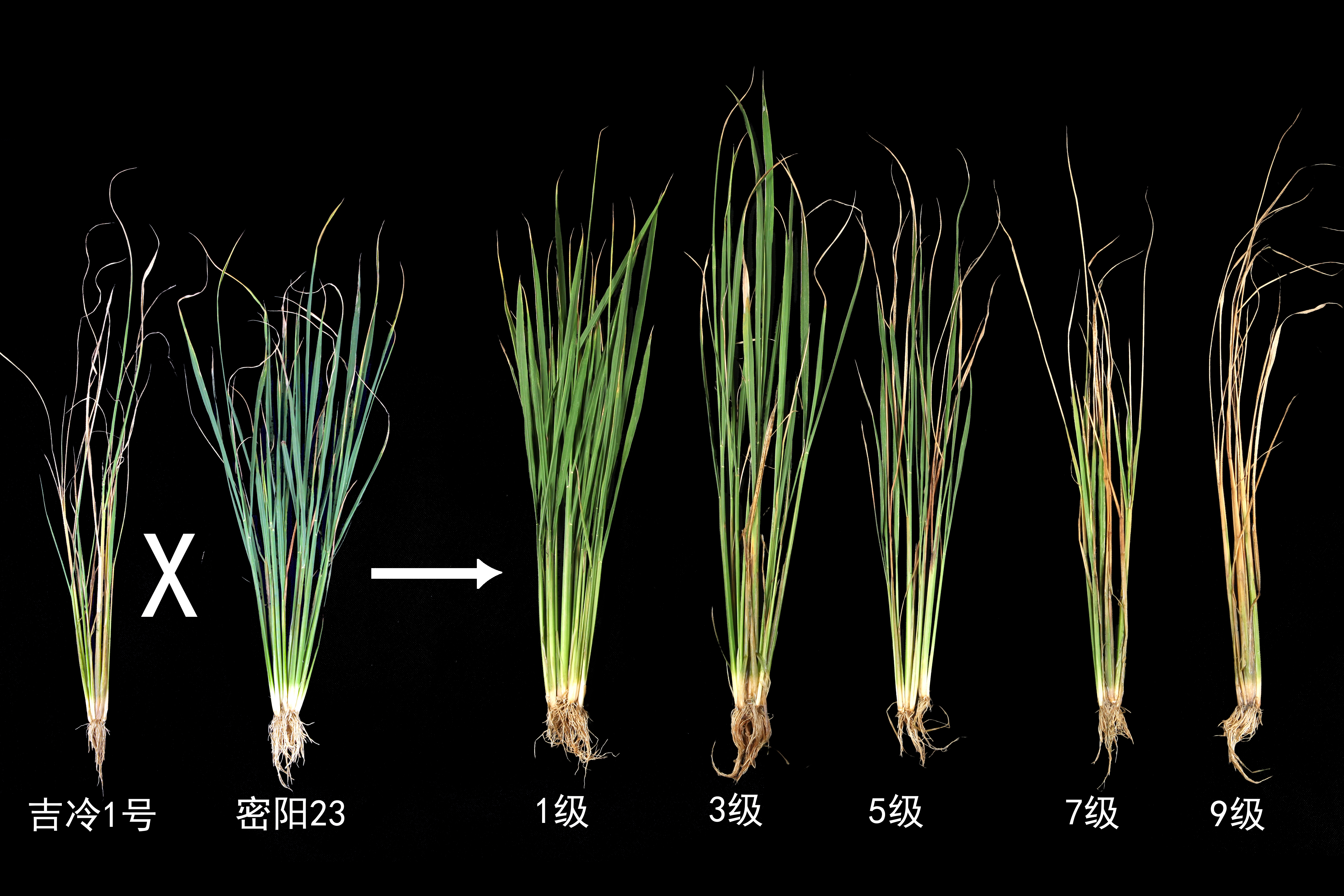

Supplement: Supplementary Figure 4 — Alignment of reference, Jileng 1 and Milyang23 for Os06g0184800 [file Image_4.jpeg]

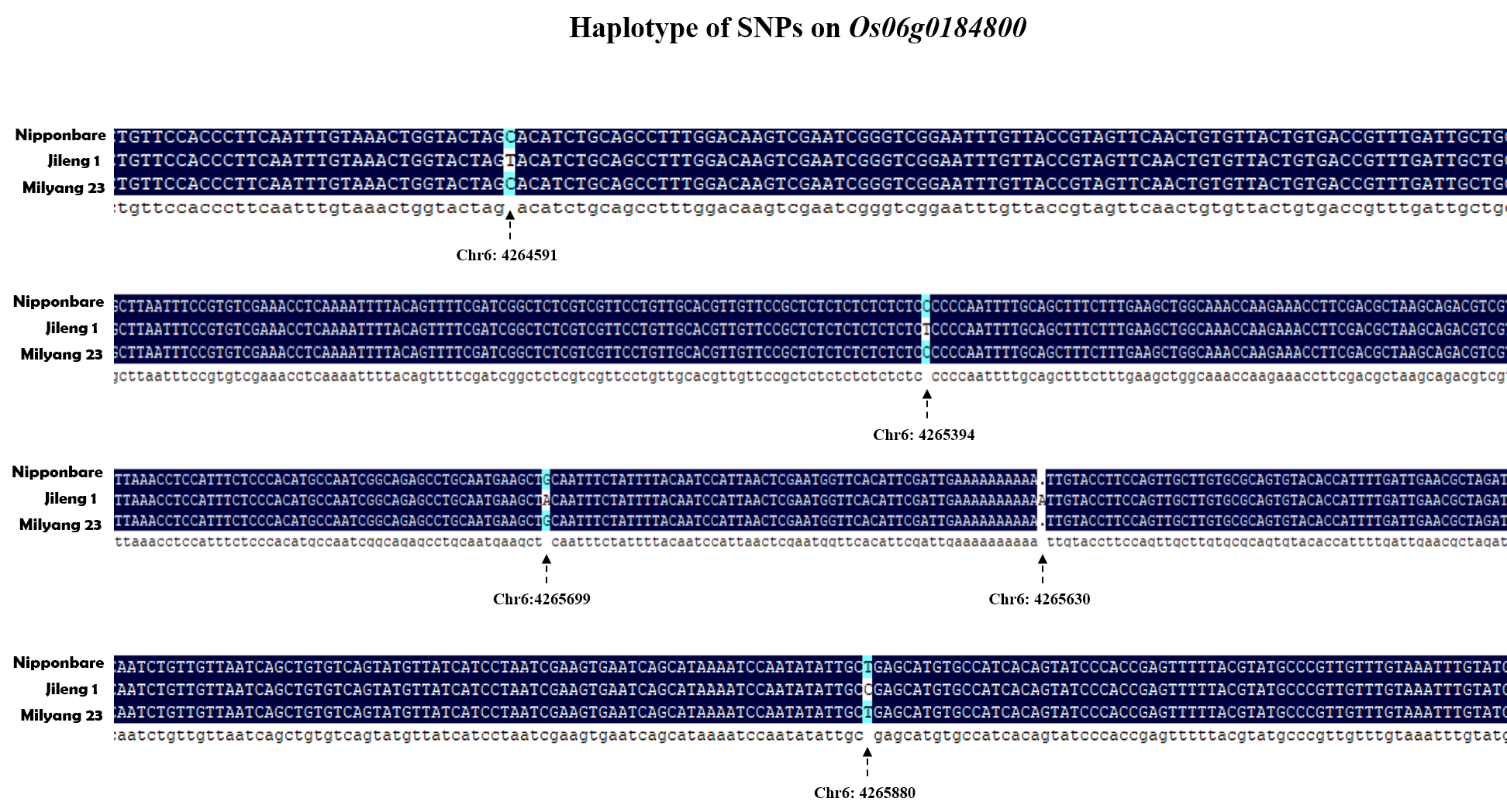

Supplement: Supplementary file 6 [file Image_5.tif]
